# Oral Colonisation after the Administration of Drops Containing *Lactobacillus reuteri*

**DOI:** 10.3290/j.ohpd.a45523

**Published:** 2020-11-20

**Authors:** Sahal Alforaidi, Andrea Bresin, Naif Almosa, Anna Lehrkinder, Peter Lingström

**Affiliations:** a Orthodontist and PhD Student, Department of Cariology, Institute of Odontology, The Sahlgrenska Academy, University of Gothenburg, Gothenburg, Sweden; Department of Pediatric Dentistry and Orthodontics, College of Dentistry, Taibah University, Medinah, Saudi Arabia. Study concept and design, collected and interpreted the data, statistical evaluation, manuscript draft, contributed substantially to discussion.; b Senior Consultant, Department of Orthodontics, Institute of Odontology, The Sahlgrenska Academy, University of Gothenburg, Gothenburg, Sweden; Specialist Clinic of Orthodontics, Public Dental Service, Region Västra Götaland, Gothenburg, Sweden. Study concept and design, proofread the manuscript, contributed substantially to discussion.; c Associate Professor, Department of Pediatric Dentistry and Orthodontics, College of Dentistry, King Saud University, Riyadh, Saudi Arabia. Study concept and design, proofread the manuscript, contributed substantially to discussion.; d Biomedical Analyst, Department of Cariology, Institute of Odontology, The Sahlgrenska Academy, University of Gothenburg, Gothenburg, Sweden. Data analysis and interpretation, proofread the manuscript, contributed substantially to discussion.; e Professor, Department of Cariology, Institute of Odontology, The Sahlgrenska Academy, University of Gothenburg, Gothenburg, Sweden. Study concept and design, ethical application, proofread the manuscript, contributed substantially to discussion.

**Keywords:** dental biofilm, dental plaque, Lactobacillus reuteri, probiotics, qPCR, saliva, Streptococcus mutans (*S. mutans*)

## Abstract

**Purpose:**

To investigate the oral colonisation potential after four weeks’ administration of *Lactobacillus reuteri* and to examine the short-term effect of probiotics on salivary *Streptococcus mutans* and lactobacilli.

**Materials and Methods:**

The study group comprised 13 young adults who volunteered after receiving verbal and written information. The short-term prospective clinical trial lasted 9 weeks, consisting of a 4-week intervention period with administration twice daily and a 5-week post-administration follow-up period with no probiotic consumption. Saliva and dental biofilm samples were obtained immediately before probiotic administration, after 2 weeks and after 4 weeks of *L. reuteri* administration. Follow-up samples were collected once every week on a regular basis after administration was terminated. The numbers of salivary *S. mutans* and lactobacilli were assessed by regular plating, while the presence of the two *L. reuteri* strains in saliva and dental biofilm was evaluated using quantitative polymerase chain reaction (qPCR).

**Results:**

The occurrence of *L. reuteri* in the oral cavity increased gradually during the intervention period and reached the maximum level after four weeks of probiotic administration (p < 0.0001). The 4-week samples of stimulated whole saliva showed a statistically significant decrease in the number of *S. mutans* and a statistically significant increase in the salivary lactobacilli level in comparison to baseline. qPCR showed that the DSM 17938 strain has better colonisation for both saliva and dental biofilm than the ATCC PTA 5289 strain at the nine-week follow-up.

**Conclusion:**

Probiotics have the ability to colonise the oral cavity during usage, but it gradually disappears after the completion of intake. It also has ability to decrease the number of salivary *S. mutans*.

Changing the microbiota to transform and maintain health is a growing issue in medical science. Probiotics have commonly been associated with gastrointestinal health, but over the last few decades, an increasing number of potential applications of bacteriotherapy in relation to oral health have also been proposed.^10^,^17^ Probiotics are defined as live micro-organisms, most often derived from the genus* Lactobacillus* (LB) or *Bifidobacterium*, which may beneficially influence gut and oral health.^6^ Probiotic bacteria can be utilised as effector strains; probiotic micro-organisms for caries prevention have been broadly evaluated. Several clinical studies have investigated the role of these probiotic micro-organisms in the inhibition of *Streptococcus mutans*, which is considered to be one of the most common caries-associated micro-organisms.^24^ The effect of an ice-cream containing *Bifidobacterium lactis* Bb-12 on the number of salivary mutans streptococci and lactobacilli in healthy adults over a shorter test period has been demonstrated,^3^ with a significant reduction in mutans streptococci after probiotic consumption. Furthermore, in a cross-over design, another study^14^ investigated the effect of *Lactobacillus reuteri* in fermented cow’s milk, showing an antibacterial effect by the tested bacteria against *S. mutans.* However, contrary results also exist, where a negative effect on *S. mutans* counts was found after 45-day administration of a mixture of lactobacilli.^13^

Colonisation in relation to the exposure time has recently attracted increasing attention.^4^,^10^ Based on saliva samples and PCR analyses, oral colonisation has been shown to be a temporary phenomenon, with survival of up to 12 days after two weeks of probiotic administration using *Lactobacillus rhamnosus* GG.^11^ Previous studies have reported different modes of probiotic administration, such as lozenges,^17^ tablets,^10^ or even in food products such as milk^20^ or yoghurt,^5^ and demonstrated their possible effect on the oral ecology. Using drops is a novel form of administration with only a few studies considering this mode as a vehicle for probiotic distribution to a group of children.^21^,^22^ Moreover, in another short-term study of a different bacterial strain (*L. reuteri* ATCC 55730), the number of probiotic bacteria gradually started to decrease shortly after the end of exposure and had completely disappeared five weeks post intervention.^4^ A variation in washout periods, ranging from two^4^ to four^16^ and six weeks,^5^ has been demonstrated. To date, there is lack of consensus on the time elapsed until oral bacterial recolonisation. The limited data available necessitate a prospective clinical study to demonstrate the time needed for the re-establishment of oral microbiota after probiotic administration. The primary aim of this study was therefore to investigate the extent to which two *Lactobacillus reuteri* strains, *L. reuteri* DSM 17938 and *L. reuteri* ATCC PTA 5289, were detectable in the oral cavity after four weeks of probiotic-drop administration. The second aim was to study the short-term effect of probiotic drops on the level of salivary *S. mutans* and lactobacilli. The null hypothesis was that the selected strains would only be detectable for two weeks after probiotic cessation and that probiotic drops would not alter the bacterial levels.

## Materials and Methods

### Participants

The sample population consisted of 13 participants, 7 females and 6 males, with a mean age of 25.7 ± 3.6 years. The power calculation was based on a 5% significance level and 80% power in order to detect a clinically relevant (40%) difference in *S. mutans* counts. The inclusion criteria were: no systemic medical condition, no medications, nonsmoker, and harbouring >10^4^ CFUs of *S. mutans* per ml saliva. The exclusion criteria were cleft lip and palate syndrome, handicapped patients, individuals with systemic diseases or conditions that could interfere with the study, and a history of probiotics/anti-inflammatory drugs/antimicrobial substances taken during the last four weeks prior to the baseline examination. All subjects had good oral health with no open or untreated caries lesions, a DMFT of 2.0 ± 1.6, and self-reported toothbrushing twice daily.

The subjects were instructed to avoid other probiotic-containing products, xylitol chewing gums and antibiotics during the study. The study protocol was in accordance with the Helsinki Declaration of Human Rights and was approved by the Ethics Committee at the University of Gothenburg (260-18). The subjects received both oral and written information about the study and gave their informed consent.

### Study Design

A short-term, 9-week prospective clinical trial was performed. It was divided into a 4-week intervention and a 5-week post-treatment period. During the intervention period, the volunteers were asked to rinse their mouths with five drops of probiotic mixed with 5 ml of distilled water in a prepared graded test tube twice a day for 60 s and then to spit it out. They were instructed to refrain from any other probiotic consumption during the entire study period. The administration of probiotics was performed in the morning, after breakfast and toothbrushing, and in the evening after brushing and before going to bed. The subjects were told to brush their teeth twice a day. For standardisation purposes, the same toothpaste (Folktandkräm, Proxident; Falun, Sweden) was distributed to all the participants.

Saliva and plaque samples were collected at baseline, at two and four weeks (at the end of intervention) and once a week during the 5-week post-intervention period. The participants were asked to refrain from proximal cleaning for 48 h and toothbrushing for 24 h prior to each sampling session. Compliance regarding the use of probiotics was checked using a special mobile app, MyMedscheduleR Plus, which reflects the percentage of mouthrinses with probiotic during the study period through a reminder given at a specific time.

### Study Drops

The probiotic drops, BioGaia Reuteri drops (BioGaia; Stockholm, Sweden), contained freeze-dried *L. reuteri* DSM 17938 (>10^8^ CFU/5 drops) and *L. reuteri* ATCC PTA 5289 (>10^8^ CFU/5 drops) suspended in oil. The daily intake was 0.15 to 0.20 g (5 drops). The probiotic solution was prepared fresh prior to each rinsing session by mixing five drops of a probiotic oil (*L. reuteri* DSM 17938 and *L. reuteri* ATCC PTA 5289) with 5 ml of water. Tubes were filled with 5 ml of distilled water and distributed to all subjects at baseline and two weeks for use. During the study, the participants were instructed to keep the water and drops in the refrigerator when not in use.

### Microbial Samples

The subjects came to the Department of Cariology, Institute of Odontology, to provide oral samples. For each individual, samples were taken throughout the study at one and the same time point. Saliva and dental biofilm were collected immediately before the start of probiotic administration (baseline), after two weeks’ and after four weeks’ administration of *L. reuteri*, as well as each week during the follow-up period (a total of sampling times).

Whole stimulated saliva (~5 ml) was collected by chewing 1 g of paraffin wax while spitting into a graduated test tube, after which the secretion rate was calculated in ml/min. One ml was transferred to VMGII medium for microbiological analysis and 1 ml was utilised to determine buffering capacity. Buffering capacity was assessed using the technique described by Ericsson^8^ and determined as final pH. One ml of saliva was placed in an Eppendorf tube and centrifuged for 10 min at 9000 rpm. The supernatant was removed and the pellet was re-suspended in TE buffer. Samples were kept at -40°C until DNA extraction for strain-specific qPCR analysis. Using a sterile toothpick, a pooled plaque sample was collected from both the maxilla and mandible to determine the probiotic strains using the qPCR technique.

### Microbiological Analysis

All samples were analysed at the Department of Cariology. The saliva samples were serially diluted (10-1 to 10-6) in phosphate buffer (PBS). Aliquots of 25 µl were then plated in duplicate on MSB (Mitis Salivarius Bacitracin) for *S. mutans* and on Rogosa agar to establish the total number of lactobacilli. Plates were incubated anaerobically in a candle jar at 37°C for two and three days, respectively.^19^ The colony count in CFU was established by identifying characteristic colony morphology.

DNA extraction from the collected biofilm and saliva pellets was performed using a GeneJET Genomic DNA Purification Kit (Thermo Fisher Scientific; Waltham, MA USA), following the manufacturer’s Gram-positive bacteria genomic DNA purification protocol. DNA concentration and purity (260/280 nm > 1.8) were measured using a Nanodrop 2000 spectrophotometer (ThermoScientific; Waltham, MA, USA) and DNA integrity was checked by electrophoresis on 1.2% agarose gel stained with GelStar (Bionordika; Stockholm, Sweden). The qPCR absolute quantification analysis was performed on an MIC analyser (Bio Molecular Systems; Upper Coomera, QLD, Australia). In total, the reaction mixture of 20 µl contained: 1x qPCRBIO SyGreen mix (PCR BioSystems; London, UK), 400 nM of each forward and reverse primer (LLC, Sigma-Aldrich; St Louis, MO, USA) and 2.5 µl (<1 µg genomic) of DNA template. All the amplifications were carried out as duplicates in MIC tubes and caps (Bio Molecular Systems). The thermocycling programme included: initial denaturation step (98°C, 2 min) with 40 cycles of denaturation (98°C, 10 s), as well as annealing plus elongation steps (60°C, 15 s). After each cycle, a ‘plate read’ step detected the increase in the fluorescence of the reporter dye (SYBR®Green, qPCRBIO SyGreen Mix, PCR Bio Systems Limited; London, UK). Finally, a melting curve analysis of PCR products was performed in the range of 70°C to 95°C to determine the specificity of the amplified products. All data were analysed using MIC software. Standard curves for the quantification of specific bacterial strains were constructed with known concentrations (10-fold dilution from 10^8^ to 10^1^ in ultrapure water) of genomic DNA extracted from reference strains. *L. reuteri* DSM17938 detection was performed with strain-specific primers: forward TTAAGGATGCAAACCCGAAC and reverse CCTTGTCACCTGGAACCACT. *L. reuteri* PTA5289 was detected with forward GACAGTGGCTAAACGCCTTC and reverse primers AATTCCACTTGCCATCTTCG.^17^,^18^

### Statistical Method

The data were processed and analysed with GraphPad Prism software (version 8.2.0 (272); San Diego, CA, USA). One-way ANOVA with Tukey’s comparison test was performed to compare different time points from the baseline to the week nine follow-up in order to detect the level of *S. mutans*, lactobacilli, and the two probiotic strains in saliva. A value of p < 0.05 was considered statistically significant.

## Results

All 13 subjects participated on all the sampling occasions (n=13). The compliance, based on the use of the specially designed app, was regarded as good (98.1%). This percentage describes the mean use of probiotics by all participants, after calculating the percentage with which each participant separately used the Plus app. The app aimed to track subjects while using probiotics and share information with the research team.

**Fig 1:**
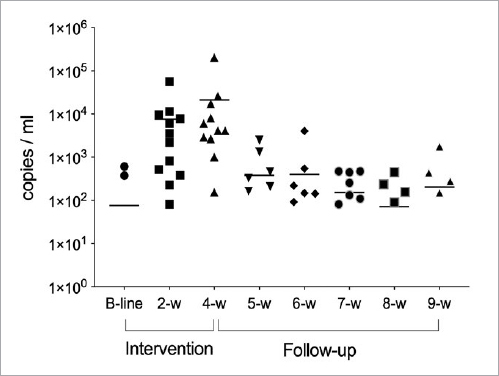
Detection of *L. reuteri* DSM 17938 in saliva via qPCR. The line indicates the mean and the symbols describe the number of subjects who are positive for the DSM 17938 strain.

**Fig 2:**
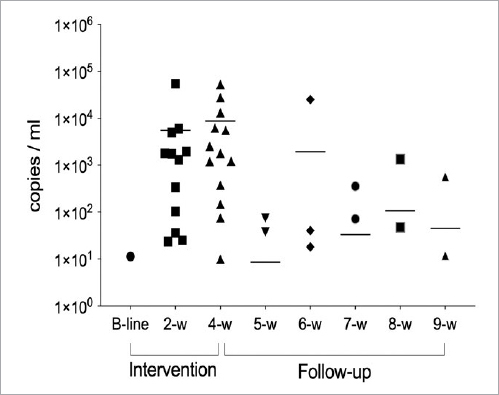
Detection of *L. reuteri* PTA 5289 in saliva via qPCR. The line indicates the mean, while the symbols describe the number of subjects who are positive for the PTA 5289 strain.

The qPCR analysis of the saliva showed that two participants carried the *L. reuteri* DSM 17938 strain, while one participant carried the *L. reuteri* ATCC PTA 5289 strain in saliva at the baseline measurement (Figs 1 and 2). After four weeks of intervention, 11 (84.6%) and 13 (100%) individuals were positive for *L. reuteri* DSM 17938 and ATCC PTA 5289 in saliva, respectively, in comparison to the baseline measurements (p<0.0001). The probiotic bacteria began to disappear gradually from the first week of follow-up, and only four individuals harboured the DSM 17938 strain and two the PTA 5289 strain five weeks after intervention. The qPCR analysis of the plaque showed a trend similar to that for saliva, but neither of the two strains was detected at baseline (Table 1). The qPCR showed that the DSM 17938 strain has slightly better colonisation features for both saliva and dental biofilm than the PTA 5289 strain at nine weeks, with 4/13 and 2/13 subjects, respectively.

**Table 1:** Positive detection of BioGaia probiotic strains in plaque samples using the strain-specific qPCR technique

Study period	*Lactobacillus reuteri* DSM 17938		*Lactobacillus reuteri* PTA 5289	
	Maxilla	Mandible	Maxilla	Mandible
Baseline	0 (0%)	0 (0%)	0 (0%)	0 (0%)
Two weeks of intervention	9 (69.2%)	12 (92.3%)	10 (76.9%)	13 (100%)
Four weeks of intervention	12 (92.3%)	13 (100%)	12 (92.3%)	12 (92.3%)
One-week follow-up	5 (38.5%)	2 (15.4%)	3 (23.1%)	3 (23.1%)
Two-week follow-up	2 (15.4%)	1 (7.7%)	3 (23.1%)	0 (0%)
Three-week follow-up	2 (15.4%)	3 (23.1%)	0 (0%)	0 (0%)
Four-week follow-up	0 (0%)	0 (0%)	1 (7.7%)	0 (0%)
Five-week follow-up	0 (0%)	0 (0%)	0 (0%)	1 (7.7%)

Number of subjects (percentage).

**Fig 3:**
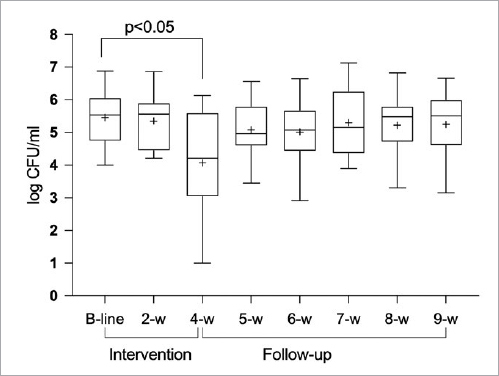
*Streptococcus mutans* prevalence in saliva. Whiskers represent max-min values; the box extends from the 25th to the 75th percentiles; the line in the middle of the box is plotted at the median; a “+” indicates the mean value.

**Fig 4:**
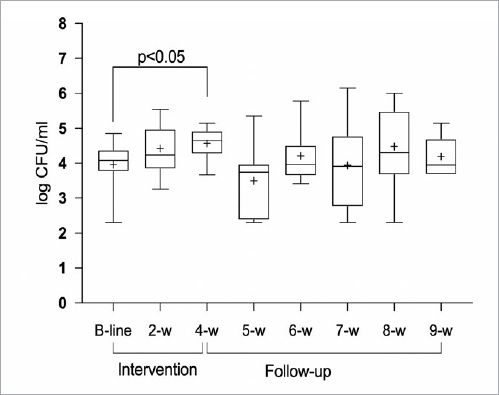
Lactobacilli prevalence in saliva. whiskers represent max-min values; the box extends from the 25th to the 75th percentiles; the line in the middle of the box is plotted at the median; a “+” indicates the mean value.

The culture analysis revealed a statistically significant reduction in the number of *S. mutans* at the end of the intervention period (p<0.05), while a statistically significant increase in the lactobacilli level was recorded (p<0.05). Apart from week 4, a similar standard deviation was observed at all time points. No statistically significant changes in the level of bacteria during the follow-up period were observed (Figs 3 and 4). The salivary secretion rate and buffering capacity revealed no statistically significant changes during the entire study period.

## Discussion

To date, studies of the oral colonisation of probiotics are limited and the subject has not been explored in any detail. The aim of present study was to follow colonisation of two *L. reuteri* strains and the effect on microbial composition. Thus, no other clinical variables were evaluated.

The main finding based on the qPCR analysis in the present study was that, when administered as a mouthrinse, both the *L. reuteri* DSM 17938 and ATCC PTA 5289 strains had the ability to be incorporated into the saliva and the oral biofilm starting from the second intervention week. It is noteworthy that a few individuals already harboured the two strains at baseline. However, they all gave an assurance that they had not used any probiotic products for the last four weeks prior to the test. It has previously been shown that individuals may harbour these strains naturally^7^,^15^ or that they may occur naturally in some food products.^9^ A variation was seen regarding the occurrence in saliva and oral biofilm, as all the subjects displayed the presence of ATCC PTA 5289 in saliva at four weeks, but only 11 did so for DSM 17938, and also their decline varied after the cessation of usage. A similar variation was seen for dental biofilm. There may be different reasons why not all individuals harboured both strains at two and four weeks. These include possible strain differences in sensitivity to oxygen exposure (facultative anaerobes) and in robustness in relation to the overall oral condition. The individual salivary and biofilm composition may also be of importance. The actual bacterial load of the probiotics and their viability at the time of mouthrinse administration was not assessed in the present study. However, pilot tests have found the information given by the manufacturer to be correct (>10^8^ CFU/5 drops). A low DMFT was seen for all individuals, and all were considered to have low caries activity.

Quite soon after the cessation of administration, a decline was seen. The total clearance of the probiotic after five weeks of follow-up from the oral cavity was noticed in most subjects for saliva, with only four (30%) and two individuals (15%) being positive with regard to the DSM 17938 and ATCC PTA 5289 strains, respectively. This indicates that no permanent colonisation had occurred either in saliva or dental plaque. This corresponds well with a previous study where it was shown that a probiotic product (bio-yoghurt) containing two different strains of lactobacilli was not able to remain in the oral cavity after one week of consumption.^1^ In addition, it has been found that after the use of probiotics containing *L. reuteri* for two weeks, the number of probiotic bacteria started to decrease gradually to 8% one week after intervention, and none of the participants harboured the strain in their saliva after five weeks.^4^ The present results thus matched the findings of previous works, in which the persistence of probiotic in the oral cavity is a temporary phenomenon and continuous administration is necessary.^1^,^11^,^12^ A similar pattern was seen for the plaque samples using qPCR analysis, although fewer individuals overall harboured the bacteria in their dental biofilm. This may be explained by the fact that other bacteria present in plaque possess antagonistic properties that prevent probiotics from colonising.

Data from most of the previously published studies have relied on the assessment of probiotic strains cultured on agar.^1^,^4^,^12^One interesting finding was that the culture analysis using selective media revealed a reduction in the salivary levels of *S. mutans* during the intervention period, followed by an increase post treatment. The corresponding data for the total number of lactobacilli in saliva showed an increase during the test period, after which the number of log CFU/ml decreased. Only the four-week samples differed significantly from baseline for both bacterial strains, indicating that it is important to allow time for the probiotics to achieve their desired effect and that continuous administration is required. The null hypothesis relating to colonisation time and the change in bacterial level was therefore rejected. No control group was included in the present study, since colonisation was followed on an individual level and it was expected that the mouthrinse was the source of *L. reuteri*. This follows a previous study design.^4^,^11^ Even if lactobacilli are known to contribute to dental caries, certain strains are known to be more related to health. There is no evidence in the literature that probiotic strains may contribute to dental caries.^23^

To our knowledge, the current study is novel, the first to investigate the adherence and the possible effect on caries-associated micro-organisms in the oral cavity using drops as a vehicle for administration of probiotics in healthy adults. The aim was to find an easily applicable mode in the oral cavity, together perhaps with better adherence features that can affect the bacterial colonies differently. Further strengths of this study are that compliance was followed using a suitably designed app, which is a problem in most clinical studies. In addition, both saliva and dental biofilm were collected and the results from regular plating as well as qPCR analysis were considered. The qPCR technique was used here in order to investigate colonisation after probiotic cessation. This technique for nucleic acid detection has been proven in terms of speed, sensitivity and specificity.^2^ Regarding the selected age group, the present study comprised young adults. However, it is possible to discuss whether a younger age group should have been the focal point, as permanent colonisation might be possible if probiotics were administerd during childhood.^11^

## Conclusion

The probiotics administered in the form of drops and used as a mouthrinse entered into both saliva and biofilm during usage, but were not able to become established in the oral cavity after a short-term exposure. They also showed the ability to decrease the number of salivary *S. mutans*. In future studies, it would be of interest to follow the effect on different caries-related variables and the actual caries outcome in individuals with varying caries activity.
